# A Series of N-of-1 Trials for Traditional Chinese Medicine Using a Bayesian Method: Study Rationale and Protocol

**DOI:** 10.1155/2021/9976770

**Published:** 2021-04-17

**Authors:** Lizhi Lu, Jiaqi An, Huijia Chen, Peilan Yang, Minhua Xu, Yingen Wu, Zhenwei Wang, Lihua Shen, Xinlin Chen, Haiyin Huang

**Affiliations:** ^1^Department of Respiratory Disease and Department of Pharmacy, Yueyang Hospital of Integrated Traditional Chinese and Western Medicine, Shanghai University of Traditional Chinese Medicine, Shanghai 200437, China; ^2^Longhua Hospital, Shanghai University of Traditional Chinese Medicine, Shanghai 200032, China; ^3^Basic Medical College of Guangzhou University of Traditional Chinese Medicine, Guangzhou 510006, China

## Abstract

*Background*. Our previous studies showed that N-of-1 trials could reflect the individualized characteristics of traditional Chinese medicine (TCM) syndrome differentiation with good feasibility, but the sensitivity was low. Therefore, this study will use hierarchical Bayesian statistical method to improve the sensitivity and applicability of N-of-1 trials of TCM. *Methods/Design*. This is a randomized, double-blind, placebo-controlled, three-pair crossover trial for a single subject, including 4–8 weeks of run-in period and 24 weeks of formal trial. In this study, we will recruit a total of 30 participants who are in the stable stage of bronchiectasis. The trial will be divided into three pairs (cycles), and one cycle contains two observation periods. The medications will be taken for three weeks and stopped for one week in the last week of each observation period. The order of syndrome differentiation decoction and placebo will be randomly determined. Patient self-reported symptom score (on a 7-point Likert scale) is the primary outcome. *Discussion*. Some confounding variables (such as TCM syndrome type and potential carryover effect of TCM) will be introduced into hierarchical Bayesian statistical method to improve the sensitivity and applicability of N-of-1 trials of TCM, and the use of prior available information (e.g., “borrowing from strength” of previous trial results) within the analysis may improve the sensitivity of the results of a series of N-of-1 trials, from both the individual and population level to study the efficacy of TCM syndrome differentiation. It is the exploration of improving the objective evaluation method of the clinical efficacy of TCM and may provide reference value for clinical trials of TCM in other chronic diseases. This trial is registered with ClinicalTrials.gov (ID: NCT04601792).

## 1. Background

### 1.1. N-of-1 Trials and Traditional Chinese Medicine

Traditional Chinese medicine (TCM) emphasizes treatment based on syndrome differentiation which is indeed the individualized treatment. It is difficult to carry out a standard form of population-based RCTs due to the individualized TCM intervention. The shortage of a reliable and evidence-based clinical efficacy evaluation method of TCM has impeded its globalization and further development [1]. Therefore, it is of great significance to explore and establish a clinical trial method that can fully reflect the individualized characteristics of TCM [2]. N-of-1 trials take the subject him(her)self as the control, and the results will be ultimately used to guide the subject's own treatment. The individualized treatment concept represented by N-of-1 trials is naturally compatible with the principle of (necessarily individually applied) syndrome differentiation of TCM, providing a feasible way for the connection between TCM and western medicine. N-of-1 trials have been listed as “level 1” evidence in the Oxford Centre for Evidence-Based Medicine 2011 levels of evidence [3] and CONSORT extension for reporting N-of-1 trials (CENT) has been published in 2015 [4]; N-of-1 trials are attracting more and more attention from scholars worldwide.

### 1.2. Bronchiectasis

Bronchiectasis is a common chronic lung disease with inconsistent clinical features and prognosis [5]. In Europe and North America, the prevalence of bronchiectasis is estimated to range from 53 to 566 per 100000 inhabitants, while in China it is approximately as high as 1200 per 100000, making it a serious and growing economic health burden [6–8]. During the stable stage of bronchiectasis, there are still clinical symptoms such as chronic cough, purulent sputum, dyspnea, or extrapulmonary symptoms such as insomnia, fatigue, constipation, or diarrhea. Western medicine lacks effective therapy for stable bronchiectasis, while TCM has good effects in alleviating symptoms and improving the quality of life [9]. At present, there is no standard TCM decoction for the treatment of stable bronchiectasis. Treatment principles are based on TCM syndrome differentiation, including resolving phlegm, clearing lung heat, and strengthening healthy energy [9]. Stable bronchiectasis is a good indication for carrying out N-of-1 trials: (1) not self-limited disease, (2) relatively stable condition, and (3) the treatment needs to be long term [4]. Yet, the requirement of N-of-1 trials is that the therapeutic drug must have a rapid onset and termination of action, and the optimal duration of treatment should be known and practical [4].

### 1.3. Our Previous Studies and Facing Problems

Since 2012, our team has been exploring the methodology of N-of-1 trials of TCM. We found this method is welcomed by patients because it embodies personalized thinking and is close to the clinical practice of TCM. In the research, we mainly faced the following two problems: (1) because of the long period (usually 3-4 weeks) of N-of-1 trials of TCM, and generally only three cycles were conducted, in terms of individual statistics, its power of statistical analysis was insufficient, which made it difficult for some patients to draw a clear conclusion [10]; (2) through a series of N-of-1 trials, we have proved that the syndrome differentiation decoction was statistically better than the fixed decoction on symptom scores (*P* < 0.05), but the result was not clinically significant. It may be related to the complex composition of TCM and its relatively long onset and failure time (half-life). The nature of TCM may not perfectly satisfy a certain requirement of the classic N-of-1 trials (rapid onset, short half-life, and fast disappearance of efficacy after discontinuation). The possible carryover effect may reduce the sensitivity of the N-of-1 trials of TCM [10]. Other investigators also believe that the carryover effect of TCM is a factor worthy of attention [11, 12].

### 1.4. Hierarchical Bayesian Statistical Method

In order to improve the efficiency of TCM N-of-1 trial design, Chen and Chen [13] tried to provide practice of guidance for the analysis of N-of-1 trials by the comparison of four commonly used models (paired *t*-test, mixed-effects model of difference, mixed-effects model, and meta-analysis of summary data). The conclusion was that mixed-effects model provided an alternative when there was carryover effect for normally distributed data of N-of-1 trials. If the factor of carryover effect is introduced into statistical models, it may improve the efficiency of data analysis of N-of-1 clinical trials for TCM.

Hierarchical Bayesian statistical method has been one of the main statistical methods for N-of-1 trials due to its significant advantages [14, 15]: (1) both individual and aggregate analyses to be simultaneously and coherently undertaken, even when the number of completed cycles between patients is variable; (2) easily introducing confounding variables, such as different patient's constitution, different TCM syndrome type, and potential carryover effect; and (3) in addition, Bayesian methods enable the use of prior available information (e.g., “borrowing from strength” of previous trial results) within the analysis, which may improve the sensitivity of N-of-1 trials [16, 17]. This method is currently rarely applied in N-of-1 trials of TCM, and it is a statistical method worthy of studying and promotion.

In view of the importance of N-of-1 trials for TCM study, we envisaged strategies to improve its sensitivity: applying hierarchical Bayesian statistical method, considering introducing the carryover effect of TCM as confounding variables into hierarchical Bayesian statistics [12, 13], and its special advantage would be used: using the data of similar N-of-1 trials that have been completed in the past as prior information [16, 17], without increasing the cycles of the N-of-1 trials, to improve the reliability and sensitivity of N-of-1 trials of TCM.

## 2. Hypothesis

The efficacy of TCM based on syndrome differentiation treatment is expected to be significantly better than that of placebo at both the individual and population levels in N-of-1 trials. But there is the possibility of invalid cases, which may be related to some factors (such as the severity of the disease, bacterial strains, and accompanying disease). Introducing carryover effect of TCM as confounding variables into hierarchical Bayesian statistics, and using the “borrowing from strength” [16, 17] function of prior information, can improve the reliability and sensitivity of N-of-1 trials of TCM.

## 3. Methods

### 3.1. Study Design

**Figure 1:**
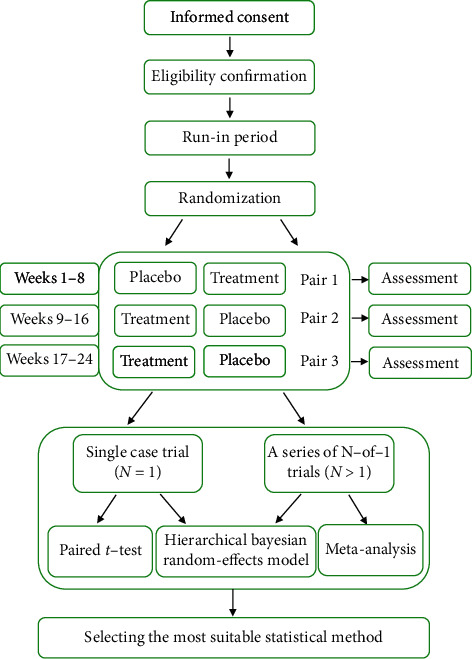
The flowchart of the N-of-1 trials in the treatment of stable bronchiectasis by traditional Chinese medicine based on syndrome differentiation.

These are a series of randomized, double-blind, placebo-controlled, N-of-1 clinical trials. According to our previous experimental design [10, 18], the brief description is as follows: we will conduct a 4- to 8-week run-in period for patients who meet the inclusion criteria to obtain onset time after drug administration and efficacy maintenance time after drug withdrawal (with the changes of patients' self-rated symptom scores as the primary outcome), so as to determine the length of observation period and the estimation of washout period. Combining the results in the run-in periods and the previous results [10, 18], we fixed the observation periods of the N-of-1 trials to four weeks. Three cycles (pairs) of N-of-1 trials will be conducted in the same individual, with each cycle consisting of two observation periods (the experimental period and the control period) assigned in random order [18, 19]. The medications will be taken for three weeks and then stopped for one week in each observation period. We will measure the outcomes in the last week of each period and the time before this is supposed to be the washout period (Figure 1). The changes in patients, self-rated symptom scores are the primary outcome (the 7-point Likert scale). Hierarchical Bayesian statistical method will be the key statistical method of this study. Different mathematical models (paired *t*-test, hierarchical Bayesian, and meta-analysis) for N-of-1 trials of TCM will be compared to improve the sensitivity and applicability of N-of-1 trials of TCM on both individual and population levels. The trial was performed at the clinic of Yueyang Hospital of Integrated Traditional Chinese and Western Medicine, Shanghai University of Traditional Chinese Medicine. This study started in January 2019 and was expected to end in December 2022.

### 3.2. Study Participants

According to our previous experimental design [10, 18], the brief description is as follows. For all patients participating in the trial, we will strictly screen according to the following criteria [10].

#### 3.2.1. Diagnostic Criteria

1. *Diagnostic Criteria of Western Medicine*. Modern medical diagnostic criteria are based on the consensus of domestic experts [5] and combined with the guidelines for the diagnosis and treatment of adult bronchiectasis published by the European Respiratory Society in 2017 [20]. All patients require a definitive diagnosis of bronchiectasis on high-resolution CT. And they should be in the stable stage of bronchiectasis, with stable symptoms such as cough and expectoration.
2. *Diagnostic Criteria of TCM Syndrome*. The diagnostic criteria of TCM syndrome are based on the “Criteria of Diagnosis and Therapeutic Effect of TCM Diseases” issued by the State Administration of TCM [21] and integrated with the TCM differentiation of bronchiectasis summarized from the literature [22], mainly including lung and spleen deficiency syndrome, qi and yin deficiency syndrome, and phlegm-heat obstructing lung syndrome (including mild phlegm-heat syndrome).

Patients who can be diagnosed with TCM syndrome should have corresponding two primary symptoms or more than two accompanied symptoms with the corresponding tongue and pulse signs.

Clinically, the TCM syndrome differentiation of stable bronchiectasis is mostly a deficiency and excess, mainly of a certain syndrome. Each type of syndrome differentiation of TCM is mixed with a certain degree of phlegm and heat. For example, qi and yin deficiency intermingled with phlegm and heat syndrome or lung and spleen deficiency with phlegm and heat syndrome [9], it is necessary to estimate the severity of intermingled phlegm and heat according to the clinician's experienced syndrome differentiation. In order to ensure the accuracy and high quality of treatment based on syndrome differentiation of TCM, the TCM syndrome of each subject will be discriminated and concluded by two chief physicians. If necessary, it will be decided by a third party (an outstanding veteran doctor of TCM).

#### 3.2.2. Inclusion Criteria

The inclusion criteria are as follows: (1) meeting the above diagnostic criteria of Chinese and Western medicine; (2) male or female, between 18 and 70 years of age; (3) being in the stable stage, and no acute exacerbation of bronchiectasis within the past three weeks; (4) frequency of acute exacerbation of bronchiectasis ≤3 times every year; and (5) signed informed consent.

#### 3.2.3. Exclusion Criteria

The exclusion criteria include the following: (1) patients who do not meet the above diagnostic and inclusion criteria; (2) suffering from respiratory failure with estimated survival time <1 year; (3) complicated with hemoptysis; (4) complicated with active pulmonary tuberculosis; (5) having pregnancy or severe heart, liver, and kidney dysfunctions; and (6) participating in other pharmacological clinical trials within the past 3 months.

#### 3.2.4. Withdrawal Criteria

Patients who meet any of the following conditions will be excluded from the study: (1) patients who voluntarily withdrew from the trial; (2) patients with poor compliance and not taking the medication as required by the study protocol; and (3) patients who are allergic to the medication.

#### 3.2.5. Participant Recruitment

The trial will be set up and coordinated by Yueyang Hospital affiliated to Shanghai University of TCM. Subjects will be recruited from the respiratory outpatient department of the hospital, or they can be recruited online or through advertising.

### 3.3. Interventions

Basic treatment will continue to be provided to all participants in the study. After assessing patients' TCM syndrome, doctors will prescribe both individualized decoction (syndrome differentiation decoction) and controlled decoction (placebo).

#### 3.3.1. Basic Treatment of Western Medicine

Basic treatment is chest physical therapy [5, 21], mainly, including postural drainage and chest percussion to assist sputum excretion. If acute exacerbation of bronchiectasis occurs, the trial should be suspended; antibiotics and other treatments will be provided conventionally according to the guidelines for noncystic fibrosis bronchiectasis [5, 23]. Other chronic diseases (such as hypertension, coronary heart disease, and diabetes) can be treated at the same time, but the usage should be relatively fixed. Patients should record the medication in detail.

#### 3.3.2. Treatment of TCM

- *Syndrome Differentiation Treatment*. The treatment based on syndrome differentiation will be conducted by chief physicians. To ensure the high quality of treatment based on syndrome differentiation, if there is any difficulty or dispute, a third party (distinguished veteran doctor of TCM) will be invited to provide consultation and guidance. It is the highly individualized treatment of TCM, the modification of Bronchiectasis Stabilization Decoction [9] (Rhizoma Fagopyri Cymosi 30 g, Radix Lithospermi 15 g, Radix Ophiopogonis 15 g, Poria 15 g, Radix Astragali 20 g, Rhizoma Bletillae 10 g, Platycodon grandiflorum 10 g, and Semen Coicis 30 g) based on syndrome differentiation. For example, for patients with lung and spleen qi deficiency syndrome, we will add Radix Codonopsis Pilosulae, Pericarpium Citri Reticulatae, and Atractylodes Macrocephala Koidz; for patients with qi and yin deficiency syndrome, we will add Radix Adenophorae, Radix Glehniae, and Radix Rehmanniae; for patients with obvious phlegm-heat syndrome, we will add Radix Scutellariae and Herba Violae. In addition, we can also adjust the individualized decoction according to the patient's other symptoms such as loss of appetite, insomnia, and constipation. In order to reflect the high flexibility of TCM, the prescription based on syndrome differentiation can be adjusted appropriately in accordance with the patient's condition changes throughout the entire study duration. The drug (in the form of TCM granules) which has passed quality inspection in line with the national norms [18] will be provided by the Sichuan New Green Pharmaceutical Technology Development Co., Ltd.
- *Placebo*. Placebo is made by dextrin, bitter agent, edible pigment, etc., and adding 5% of the test drug. There will be no difference between the placebo and the test drug in terms of dosage form, appearance, color, specification, label, and so forth [24]. The individualized decoction and placebo will be used in the same way: take one decoction a day in two doses.

### 3.4. Randomization and Blinding

According to our previous experimental design [10, 18], the brief description is as follows: a specialized pharmacist will use a computer (software SPSS 15.0) to generate random numbers and random number sequences to determine the order of medication for each subject in each observation period, such as BA-AB-BA or AB-BA-BA. The pharmacist will use the method of flipping a coin to determine which of A or B represents individualized prescription or control prescription, record the blind code, and keep it properly. After evaluating the patient's TCM syndromes, the doctor will prescribe both individualized prescription and control prescription. Then, the two prescriptions will be delivered to the specialized pharmacist by the TCM Pharmacy. Then, the pharmacist will prepare the drug following the randomized medication order and the blind code.

### 3.5. Outcome Measures

The investigators will see the patients and collect data before and after each treatment period. Patients should identify the symptoms that bother them and make a self-administered patient diary or questionnaire. In addition, it is necessary to establish online data collection approaches (WeChat, including personal contact and specialized research group chat) with the subjects in order to obtain their information conveniently and timely. Subjects can report online changes in their condition or adverse reactions during the trial in time. Meanwhile, the personal privacy of the subjects will be protected from disclosure. The following are the outcome measures.

#### 3.5.1. Primary Outcome

*Patient Self-Rated Symptom Score*. Patients should record their symptoms (cough, expectoration, shortness of breath, chest pain, loss of appetite, fatigue, insomnia, etc.) in a diary and rate the severity of these problems on the 7-point Likert scale supplemented by Visual Analogue Scales (VAS) [10, 25]. The number of questions must be optimized to ensure that the most important aspects of the patient's problem are examined (usually four to eight items). The higher the score, the more severe the symptom. Take cough as an example.

On average, in comparison with your usual cough, how severe is the cough?

1. No cough, or as mild as, or milder than they have ever been
2. Not nearly as severe as usual
3. Not as severe as usual
4. As severe as usual
5. Severer than usual
6. Very severe, almost as severe as they have ever been
7. Very severe, as severe as or more severe than they have ever been

An improvement of more than 0.5 points for a problem is considered clinically effective for this problem; if the average improvement of the total score of all symptoms is more than 0.5 points, it can be considered clinically effective for this case [19, 25]. Therefore, the mean difference of 0.5 points is defined as the “Minimal Clinically Important Difference (MCID)” for the 7-point scales.

#### 3.5.2. Other Outcomes

1. *24 h Sputum Volume*. We will measure the 24 h sputum volume and take the mean value for the 3 consecutive days at the beginning and the end of each trial. To ensure the precision of the measurement, patients are required to spit their sputum into a calibrated collector from 8:00 am to the next 8:00 am. We will use the mean value of the sputum volume for 3 consecutive days as the outcome.
2. *COPD Assessment Test (Chronic Obstructive Pulmonary Disease Assessment Test, CAT)*. In recent years, foreign scholars [26] have confirmed that the CAT is valid and reliable in patients with bronchiectasis and other chronic respiratory diseases. CAT questionnaire consists of 8 items. Each item is rated on a scale of 0–5 points, thereby making the total score to be 0–40 points. The lower the score, the better the patient's health and quality of life. The “Minimal Clinically Important Difference (MCID)” for the CAT has not been established officially, but it was estimated to be around 2 points [10, 27].
3. *Safety Outcome*. Blood routine, urine routine, liver and kidney function, electrocardiogram, and other laboratory tests, as well as some vital signs, will be determined and recorded before and after the study. We will observe whether there are any adverse reactions or events related to the test method or drug. If necessary, we will terminate and unblind the trial, provide corresponding treatment measures, and report the adverse event to the ethics committees.

### 3.6. Data Analysis

#### 3.6.1. Sample Size Calculation

Estimation of the needed sample size was based on the result of the preliminary study. The primary outcome of the study is patient self-reported symptoms scores on a 7-point Likert scale. The mean difference between the symptoms scores of the two groups of the preliminary study was 0.47, with the standard deviation (SD) being 0.53. The basis for estimating the required sample size is that it has at least 80% power (*β* = 0.20) to detect the mean difference of 0.47 points, with significance testing at the *α* = 0.05 level and SD = 0.53; using a two-sided test, the ratio of the two groups is 1 : 1, with three cross-overs, assuming no period effect or treatment × time interaction, under the given model parameters [28, 29]. The sample size was calculated by using PASS 11.0 software (NCSS LLC, Kaysville, UT, USA). Preliminary calculations concluded that 21 patients would be needed to meet the requirements. Taking into account the high drop-out rates of N-of-1 trials (30%), the final sample size was determined to be 30.

#### 3.6.2. Statistical Analysis

To avoid or decrease the potential carryover effect of the previously used drug, the values of the outcomes will be measured in the last week of each observation period. All patients with at least one complete treatment cycle will be included in the analysis. The data generated in this study will constitute a dataset, and the database will be established by experts specializing in statistical analysis of N-of-1 trials. For all data in this dataset, carryover effect, period effects, and autocorrelation will be carried out and analyzed by statistical experts [4, 10].

Hierarchical Bayesian statistical method will be the key statistical method of this study. It is based on the Monte Carlo technology of Markov's Bayesian hierarchical model calculation chain and uses WinBUGS software for calculation. The advantages of a Bayesian approach over normal frequentist statistical methods are discussed in detail in Zucker et al., Schluter and Ware, and Nikles et al. [30–32]. We intend to use the advantages of Bayesian analysis: (1) various levels and confounding variables can be introduced, such as the potential carryover effect of TCM, the severity of bronchiectasis, sputum culture (whether *Pseudomonas aeruginosa* is positive), and TCM syndrome differentiation (lung and spleen deficiency syndrome, qi and yin deficiency syndrome, phlegm-heat syndrome (mild, moderate, severe, etc.)), and the difference in efficacy under stratification and confounding variables can be analyzed. Using the data of similar N-of-1 trials that have been completed in the past as prior information, from the Bayesian analysis we will obtain posterior distributions for the mean treatment effect at the population level, as well as posterior distributions for the treatment effects at the individual level, that will exhibit borrowed strength from the population estimates through shrinkage to the population mean. The probability distributions of each model will be evaluated to assess violations and data transformations undertaken, where necessary. Conventional burn-in periods, model convergence and stability diagnostics, and residual checks will be employed.

The forms of hierarchical Bayesian statistics analysis results will be as follows: (1) the mean of the posterior distribution of the mean difference between the outcomes of placebo and the test drug, which gives the best estimate of the difference in the overall effect size between the treatments; (2) the relevant 95% credible region, which gives uncertainty interval of the posterior distribution (2.5 and 97.5 percentiles in this case), and (3) the posterior probability of mean difference that the test drug will be better than the control drug, which describes the likelihood that the patients will tend to actively treat in future cycles. When these estimated values exceed predetermined threshold values (for example, the improvement of the average of the total points of all symptoms is ≥0.5 points), the patient will be defined as a “responder” [33, 34].

To compare the novel methodology of aggregated N-of-1 trials with the traditional analysis methods, the Bayesian method will be compared with other common statistical methods used in N-of-1 trials (including paired *t*-test and meta-analysis) to evaluate its sensitivity and applicability to N-of-1 trials of TCM.

## 4. Discussion

Our previous studies [10] showed that N-of-1 trials could reflect the individualized characteristics of traditional Chinese medicine (TCM) syndrome differentiation with good feasibility and compliance, but the sensitivity was low. We supposed that the improvement of statistical models may be beneficial in establishing a clinical efficacy evaluation method that meets the characteristics of TCM.

For the past few years, hierarchical Bayesian statistical method has been widely used in this field. Senior et al. [14] concluded that the advantages of Bayesian method over ordinary frequentist statistical methods, such as the following: (1) both individual and aggregate analyses are simultaneously and coherently undertaken, (2) natural hierarchies and serial correlations within the study can be exploited and accommodated (such as clustering by physician, setting, or location), (3) confounding variables can easily be introduced into Bayesian model, and (4) they naturally allow incorporating any relevant trial information that may be sourced from elsewhere [14]. In addition, Bayesian methods allow for the use of prior available information (e.g., previous trial results) within the analysis. In the hierarchical Bayesian model, previous data and new trial data belong to a continuous data chain. When new trial data are generated, the data chain is updated to generate more accurate posterior information [15].

Bayesian methods as yet have not been used in N-of-1 trials of TCM. In this trial design, we consider introducing the carryover effect of TCM as confounding variables into hierarchical Bayesian statistics [12, 13] and using the data of similar N-of-1 trials that have been completed in the past as prior information, without increasing the cycles of the trials, by “borrowing from strength” from the results of other similar N-of-1 trials to improve the reliability and sensitivity N-of-1 trials of TCM. In addition, the Bayesian method will be compared with other common statistical methods used in N-of-1 trials (including paired *t*-test and meta-analysis) to evaluate its sensitivity and applicability to N-of-1 trials of TCM.

It should be mentioned that this trial protocol has other characteristics: (1) a relatively reasonable observation and washout period were determined through the run-in period combined with investigators' clinical experience [10, 18]; (2) placebo control used in the study has high test sensitivity, can detect the absolute effectiveness and safety of the test drug, and has a high ability to reduce bias; the carryover effect of TCM can also be calculated from it; (3) highly individualized treatment based on syndrome differentiation: not only can the investigational drug be individualized, but it can also be adjusted according to the patient's condition and syndrome changes during the whole process of N-of-1 trials, which conforms to the principle of “do what is proper according to the circumstances” in TCM. Meanwhile, the control drug will be always unchanged. Our ultimate goal is to explore the establishment of an objective evaluation method of therapeutic effects reflecting the essence of TCM treatment based on syndrome differentiation in line with the trend of international medical development. In a broad sense, it may have reference value for clinical trials of TCM for other chronic diseases.

## Data Availability

The data used to support the rationale and protocol of this study are included within the article and the protocol has been registered in ClinicalTrials.gov (no. NCT04601792). All data contained in this study can be obtained by contacting the corresponding author upon request.
